# Foreword

**DOI:** 10.3402/gha.v6i0.20446

**Published:** 2013-02-25

**Authors:** Lars Weinehall

**Affiliations:** Head of Epidemiology and Global Health, Umeå University, Umeå, Sweden

Bilateral research co-operation and research training between Vietnam and Sweden, aimed at focusing on important development issues, began in the 1970s. Today, global health and health impacts of climate change are on top of the agenda:

- Vietnam is experiencing a double burden of diseases, including both infectious (HIV/AIDS, bird flu, dengue fever, food poisoning, etc.) and non-communicable diseases (cancer, cardiovascular diseases, etc.), and is predicted to be greatly impacted by climate change in the future.
- Sweden also faces significant health challenges, in terms of chronic diseases, and will probably at the same time – like many similar countries in the West – become greatly affected by climate change.

Research cooperation between the two countries is essential to develop common knowledge and to find strategies to move from words to action. Therefore, the collaborative research spanning more than 10 years between the Hanoi Medical University (HMU), Vietnam, and Epidemiology and Global Health (EGH), Umeå University, Sweden, plays an important role. Over the years, a number of staff members from EGH have visited Hanoi as teachers, while HMU researchers have lectured in Umeå.

Scientific workshops have been conducted both in Vietnam and Sweden, and more than 20 staff members from HMU have completed their PhD or Master of Public Health at Umeå University. Some have also spent postdoctoral periods in Umeå. This cooperation has produced more than 60 scientific papers that have been jointly published in international peer-reviewed journals.

This supplement represents a new milestone in the long-term cooperation between HMU and EGH. Supported by funding from the Swedish International Development Cooperation Agency (SIDA), 15 scientific papers have been published, with special focus on how new scientific knowledge can be transformed into policy, thereby helping to improve people's living conditions. The studies represent important steps in our collective effort to implement public health research in public health policy and thereby, hopefully, reduce the know–do gap.

*Lars Weinehall* Head of Epidemiology and Global Health Umeå University Umeå, Sweden

